# Well-Being and Fear of Missing Out (FOMO) on Digital Content in the Time of COVID-19: A Correlational Analysis among University Students

**DOI:** 10.3390/ijerph18041974

**Published:** 2021-02-18

**Authors:** Ceren Hayran, Lalin Anik

**Affiliations:** 1 Marketing Department, School of Business, Ozyegin University, Nişantepe Mah, Orman Sok., Çekmeköy, 34794 Istanbul, Turkey; 2 Marketing Area, Darden School of Business, University of Virginia, 100 Darden Blvd, Charlottesville, VA 22903, USA; AnikL@darden.virginia.edu

**Keywords:** fear of missing out (FOMO), fear of missing out, COVID-19, SARS-CoV-2, pandemic, well-being, digitalization, digital content, social media

## Abstract

The majority of research on the fear of missing out (FOMO) has focused on understanding how social media posts about attractive unattended experiences taking place in the physical world (e.g., a friend’s vacation) influence individuals’ affective states. With quarantine measures in place, and in the absence of travel and party photos on social media, do individuals feel they are missing out on enjoyable experiences? The current work shows that FOMO has not disappeared during the pandemic, even when socially distancing at home, but has been replaced by feelings towards new online activities (e.g., online concerts, virtual gatherings). As a consequence, we find that FOMO threatens well-being by causing important psychological and health issues, such as sleep deprivation, loss of focus, declined productivity, and finding relief in knowing that others have difficulty keeping up with abundant digital content. Importantly, we find these consequential effects both during the initial (May 2020) and late stages (December 2020) of the pandemic. With excessive Internet use and virtual FOMO likely to be a continuing reality of life, questions remain as to how one can refrain from its negative effects and stay healthy during the pandemic and in the post-pandemic era. We discuss remedies and suggest new research avenues that may help elevate the negative consequences of FOMO on well-being.

## 1. Introduction

At the beginning of 2020, the World Health Organization (WHO) declared the outbreak of Coronavirus (COVID-19) to be a global public health emergency. It has affected every aspect of life, leading to increased poverty, unemployment, inequality, movement restrictions and an unprecedented burden on healthcare [1]. In addition to social and economic disruption, the pandemic has also created less visible yet important emotional and mental health effects. All parties, including public health authorities [2], government entities [3] and scholars (e.g., [4]), have joined forces to identify and cope with the adverse effects of this pandemic on individual and societal well-being.

One of the key preventative measures used globally is physical distancing from others in order to limit the spread of the coronavirus by close contact among individuals. Authorities have imposed diverse distancing interventions, varying from strict lockdown orders to public-gathering restrictions to limit the transmission of the disease. These measures led to decreased outdoor time and increased digital usage and screen exposure. New forms of virtual and digital working, shopping, and socializing routines have emerged. People have turned to screens and technology to meet their social needs. Social media platforms have incurred radical jumps both in the number of visits and visitors, and internet traffic increased up to 70% in the initial phase of the pandemic [5]. On one hand, this increased engagement in the online world during the pandemic may facilitate positive experiences such as helping people stay informed, feel entertained, and cope with stress [6]. On the other hand, this unprecedented rise in engagement with online tools may threaten mental well-being by increasing internet addiction disorders [7], creating anxiety and depression due to excessive exposure to negative news [8], and potentially exposing younger individuals to violent or harmful content [9]. Contributing to this important line of work, we examine the fear of missing out (FOMO) as a byproduct of excessive internet and social media usage, and test its consequences on well-being.

FOMO is defined as “a pervasive apprehension that others might be having rewarding experiences from which one is absent” [10]. More broadly, it refers to the negative feeling that results from being aware of unattended experiences. As reflected by the mounting media mentions [11] and academic work (see [12] for a review), FOMO has become pervasive in society. Supporting the ubiquity of FOMO in society, a recent study conducted before the pandemic with 936 individuals with different socio-demographic backgrounds revealed that FOMO was experienced at least occasionally by 81% of the participants [12]. Especially with the increased use of digital tools and social media, individuals are often trapped in this feeling that others are living better lives and having more enjoyable moments. With quarantine measures in place, and in the absence of travel and party photos on social media, have consumers stopped worrying about missing out on enjoyable experiences? If FOMO still exists, what are its consequences for individual well-being and how can we recover from its effects to be ready for a healthy post-pandemic era? In the current work, we seek answers to these questions in order to contribute to the understanding of how our affective and physical experiences have changed during the pandemic due to increased exposure to digital content, as well as suggest solutions for potential future crises.

While earlier work examined FOMO as a trait variable [10], more recent studies explored the extent to which situational cues at a particular moment, such as checking social media posts, could trigger FOMO [12,13]. One common characteristic of the existing work is that it addresses FOMO as a feeling that results from being exposed to information on social media about desirable offline activities. In other words, individuals experience FOMO through digital tools that inform them of attractive experiences taking place in the physical world. For example, Facebook photos or Instagram stories reflecting others’ joyful moments of travelling, partying or having fun at a music festival may trigger FOMO for those who view these posts but cannot engage in the activities. To our knowledge, no prior work has explored whether virtual experiences such as online cooking classes, Clubhouse conversations, Instagram concerts or newly released Netflix movies also trigger FOMO. Distinct from the existing work, our research focuses on understanding how FOMO has changed during the COVID-19 pandemic, when people limited their outside activities and instead engaged heavily in online experiences. Unlike offline experiences, virtual activities don not require one’s physical presence—they may seem easier to engage in and hence less likely to trigger FOMO. However, increasing digitalization and the abundant amount of online content during the pandemic makes it increasingly more difficult to catch up with a range of novel virtual experiences.

In addition to exploring whether FOMO still exists in this new landscape of online consumption, we are also interested in examining its consequences for well-being. Existing work on the correlates and outcomes of FOMO reveal that experiencing FOMO may lead to severe psychological and health consequences such as stress, anxiety [13,14], depression [15], poor sleep [16] as well as academic demotivation [17], problematic mobile phone use [18], and problematic social media use [19]. More recent work reveals that FOMO can also take the joy out of a moment and may lead individuals to report lower satisfaction with the current experience [12]. Interestingly, these effects hold even during highly pleasing activities. Expanding on this work, our goal here is to explore potential well-being effects of FOMO as an experience triggered by the increased consumption of digital content caused by the pandemic. In general terms, well-being refers to feeling good and judging life positively and indicates a broad category of people’s affective, psychological, physical and social life satisfactions [20,21]. In this work, we are specifically interested in understanding how FOMO on the abundant digital content influences individuals’ affective and physical daily experiences such as their ability to keep up with everyday routines, focusing on and enjoying their moment, sleeping, as well as their feelings about other individuals possibly experiencing similar difficulties.

We also explore how FOMO is related to the level of information seeking and sharing about pandemic. Recent research shows that COVID-19 information seeking on digital media increases individual risk perception and preventive measures on one hand, but causes intense worry and distress on the other [22,23]. We investigate if FOMO-prone individuals have been eager to search for more information and share it with others across different stages of the pandemic. It is important to understand the factors influencing risk perceptions during the pandemic as individuals with low levels of perceived risk have been shown to threaten the lives of others by failing to engage in protective behaviors such as social distancing and hand hygiene [24,25,26]. Based on these findings, we investigate if risk perception is related to individual FOMO experiences or information-seeking tendencies at the early stage of the pandemic.

Next, we present two studies conducted with university students: one during the peak of the pandemic (May 2020), and a second one during the later stage of the pandemic when the vaccine was approved and announced (December 2020).

## 2. Materials and Methods

### 2.1. Study 1—Early Stage of the Pandemic

#### 2.1.1. Participants and Data Collection Procedure

Study 1 aims to understand (1) the relationship between exposure to digital content and FOMO, (2) the relationship between FOMO and well-being, at the early stage of the pandemic, respectively. The data was collected during the spring semester of the 2019–2020 academic year, specifically during the second week of May. At this point, the COVID-19 outbreak had been present since mid-March, for one and a half months. Specifically, authorities had imposed social distancing and self-isolation warnings to minimize interactions among people, and occasional weekend lockdowns were in effect.

One hundred and seventy-eight undergraduate students at a European university (Mage = 21.35, SD = 1.82, 38% female) participated in the study in exchange for course credit. Survey link was shared with the students via e-mail, and they completed the study in their own time. Participants were informed that participation was voluntary, they could withdraw from the study, and the answers would remain anonymous. We received verbal consent from all participants before data collection. 

#### 2.1.2. Measures

Initially, participants responded to several trait measures by indicating their agreement with a series of statements on a seven-point scale (1 = not at all, 7 = very much). We administered the ten-item FOMO trait scale [10] to assess individual tendency to experience fear of missing out that others might be having rewarding experiences from which one is absent. Along with FOMO, we investigated whether extroversion, curiosity and productivity orientation as personality traits had any effect on whether people experienced fear of missing out on the digital content during the pandemic. We used the two-item shortened extroversion scale [27] to assess how outgoing and extrovert the individuals were. Additionally, we used the two-item shortened curiosity scale [28] and the four-item productivity orientation scale [29] to assess curiosity and individual tendency to be productive and accomplish more in less time, respectively.

Next, we measured FOMO as a state variable to examine to what extent participants felt that they were missing out on existing virtual social activities (“To what extent do you feel like you are missing out on ongoing virtual activities and experiences that take place in your environment?”; 1 = not at all, 7 = very much; [12]). To understand the relationship of FOMO with various digital content, we asked participants if and how much they experienced FOMO while trying to keep up with the following activities: real-time virtual events such as live concerts, interviews and sports activities, newly released movies and series, virtual gatherings with family and friends, social media posts of others, and pandemic related news. Importantly, we identified these virtual experiences, and the behavioral correlates according to the exploratory discussions we held with students. Participants then indicated how many times they engaged in online social activities (such as virtual gatherings with family and friends, watching real-time Instagram concerts or interviews) since the beginning of the pandemic. They also indicated whether the time they spent on social media increased during the pandemic, on a seven-point scale (1 = not at all, 7 = very much).

Then, we examined the correlates of FOMO regarding individual well-being. Participants were asked to indicate if they experience the following incidents when thinking about the existing virtual activities: staying up late and feeling sleep-deprived, having difficulty in fulfilling daily responsibilities, lack of concentration during the day, having difficulty to enjoy the moment, and finding relief in the idea of others’ having a hard time keeping up with the digital content, (1 = not at all, 7 = very much).

Finally, we explored how FOMO was related to an individual’s information-seeking and sharing tendencies. To understand COVID-19 information seeking tendency, participants were asked to take a few minutes to scan pandemic related news on social media, and write down three pieces of information that was new to them. Afterwards, they were asked whether they would want to continue reading the news and share what they read with others. To understand if information-seeking tendency was related to an individual’s risk perceptions, we asked participants how likely that someone they knew could get infected (What is the likelihood that someone you know may catch coronavirus?”; 1 = very low, 7 = very high). Participants further indicated whether the time they spent on reading news increased during the pandemic, (1 = not at all, 7 = very much).

#### 2.1.3. Data Analysis and Results

In the present study, statistical analyses were performed using SPSS version 20.0 for Windows (SPSS Inc., Chicago, IL, USA). Pearson’s correlation coefficient, r, was used to evaluate the association between the level of FOMO and the explored variables.

- FOMO and digital content:

Reliability index (Cronbach’s α coefficient) for FOMO trait, extroversion, curiosity and productivity orientation were 0.82 and 0.85, 0.83, and 0.78, respectively, indicating good internal consistency. Correlational analyses revealed that trait FOMO and state FOMO were positively correlated (r = 0.37, *p* < 0.001), suggesting that individuals who had a general tendency to experience fear of missing out also experienced it strongly towards digital content during the early stage of the pandemic. Importantly, participants’ actual rate of engagement in online activities (such as virtual gatherings with family and friends, and watching real-time Instagram concerts or interviews) since the beginning of the pandemic was highly correlated both with their trait FOMO (r = 0.18, *p* < 0.05) and state FOMO (r = 0.41, *p* < 0.001). This suggests that higher fear of missing out on the existing virtual social activities leads to higher engagement in them, which feeds into experiencing further FOMO.

Both trait FOMO and state FOMO were strongly correlated with the explored digital content. Specifically, they were positively correlated with missing out on real-time virtual events such as live concerts, interviews and sports activities (r = 0.30 and r = 0.30, *p* < 0.001), newly released movies and series (r = 0.27 and r = 0.20, *p* < 0.001), virtual gatherings with family and friends (r = 0.51 and r = 0.26, *p* < 0.001), social media posts of others (r = 0.40 and r = 0.35, *p* < 0.01), and pandemic related news (r = 0.28, *p* < 0.001 and r = 19, *p* < 0.05). However, other trait variables (extroversion, curiosity and productivity orientation) were not significantly correlated with any of the virtual activities (all *p*’s > 0.05). In other words, while those who had high levels of dispositional and situational FOMO experienced that they were strongly missing out on virtual activities; extroversion, curiosity and productivity orientation as individual characteristics were not related to experiencing missing out on these activities. Descriptive statistics and correlation coefficients are presented in Table 1.

- FOMO and well-being effects:

Regarding the well-being consequences, both trait FOMO and state FOMO were positively correlated with feeling sleep-deprived (r = 0.29 and r = 0.21, *p* < 0.01), having difficulty in fulfilling daily responsibilities (r = 0.32 and r = 0.23, *p* < 0.01), lack of concentration during the day (r = 0.32 and r = 0.18, *p* < 0.01), having difficulty to enjoy the moment (r = 0.44 and r = 0.37, *p* < 0.001) and finding relief in the idea of others’ having a hard time keeping up with the digital content (r = 0.47 and r = 0.26, *p* < 0.001). These strong correlations indicate that participants who had high levels of dispositional and situational FOMO experienced negative affective and physical consequences when following digital content during the pandemic. Descriptive statistics and correlation coefficients are presented in Table 2.

In addition, both trait FOMO and state FOMO were positively correlated with increased time spent on social media (r = 0.36, *p* < 0.001 and r = 0.16, *p* < 0.05) and following the pandemic related news (r = 0.20, *p* < 0.01 and r = 0.15, *p* < 0.05), indicating that higher FOMO led to spending significantly more time on both social media and reading the news during the early stage of the pandemic. Also, more curious individuals reported spending more time on social media and also in reading the news at this stage (r = 0.17, *p* < 0.05 and r = 0.26, *p* < 0.001).

- FOMO and tendency to seek and share information:

Finally, our investigation of the relation between FOMO as a personality trait and individual information-seeking and sharing tendencies revealed significant and positive correlations. Upon checking pandemic related news, participants with higher trait FOMO wanted to keep on reading the news and share what they read with others at a higher degree (r = 0.34 and r = 0.32, *p* < 0.001). Curious individuals were also more inclined to continue reading and sharing the news with others (r = 0.15, *p* < 0.001 and r = 0.32, *p* = 0.05). Hence, individuals with higher dispositional FOMO and curiosity had a higher tendency to seek and share information at the early stage of the pandemic (see Table 2). Regarding participants’ risk perceptions, we did not find any significant relations between risk perceptions and information-seeking tendencies (r = 0.06, *p* > 0.05). However, participants who had higher risk perceptions also experienced higher difficulty in following pandemic related news (r = 0.15, *p* = 0.05), but not other virtual activities. This suggests that in the early stage of the pandemic, an individual’s perceived risk of an acquaintance getting infected was positively associated with their feelings of missing out on pandemic updates.

### 2.2. Study 2—Late Stage of the Pandemic

#### 2.2.1. Participants, Data Collection Procedure and Measures

Study 2 aims to understand (1) the relationship between exposure to digital content and FOMO, (2) the relationship between FOMO and well-being at the late stage of the pandemic, respectively. The data for the second study was collected during the fall semester of the 2020–2021 academic year, specifically the third week of December. This was nine months into the pandemic when movement restrictions in the country were still in place. Specifically, authorities were imposing social distancing and self-isolation warnings and occasional weekend lockdowns were in effect. Distinct from the first study, this was towards the end of the pandemic; vaccinations were approved and announced to be distributed within a few weeks. To be able to compare the results of the two studies, the participant selection method and respondent characteristics were kept similar. Data collection procedures and the measures used were same as in Study 1.

Two hundred and fifteen undergraduate students at a European university (Mage = 21.57, SD = 1.56, 51% female) participated in the study in exchange for course credit. Participants completed the self-report survey in their own time.

#### 2.2.2. Data Analysis and Results

Reliability index (Cronbach’s α coefficient) for FOMO trait, extroversion, curiosity and productivity orientation were 0.80, 0.82, 0.65. and 0.71, respectively, indicating good internal consistency.

- FOMO and digital content:

Correlational analyses revealed that trait FOMO and state FOMO were positively correlated (r = 0.36, *p* < 0.001). Participants’ actual rate of engagement in online social activities since the beginning of the pandemic was significantly correlated with their trait FOMO (r = 0.13, *p* = 0.05) and state FOMO (r = 0.38, *p* < 0.001). Parallel with the results of Study 1, higher fear of missing out on the existing virtual social activities correlated significantly and positively with higher engagement in them, which may have led to experiencing further FOMO.

Regarding the digital content, both trait FOMO and state FOMO were positively correlated with missing out on real-time virtual events such as live concerts, interviews and sports activities (r = 0.34 and r = 0.37, *p* < 0.001), newly released movies and series (r = 0.33 and r = 0.33, *p* < 0.001), virtual gatherings with family and friends (r = 0.39 and r = 0.42, *p* < 0.001), and social media posts of others (r = 0.43 and r = 0.40, *p* < 0.001). However, trait FOMO was not related to following pandemic related news at this stage (r = 0.13, *p* > 0.05), while state FOMO was still significantly related (r = 29, *p* < 0.001). These findings show that participants with high levels of dispositional and situational FOMO still experienced strong feelings of missing out on digital content at this late stage of the pandemic. The only difference from the early stage was that FOMO-prone individuals did not feel that they were missing out on following news on the pandemic at this point in time, presumably because they might have been fed up with pandemic related information. Correlation analyses between the virtual activities and other trait variables (extroversion, curiosity and productivity orientation) revealed that only curiosity was significantly correlated with feelings of missing out on following pandemic related news (r = 0.13, *p* < 0.05) and on virtual gatherings with family and friends (r = 0.19, *p* < 0.01; see Table 1).

- FOMO and well-being effects:

Concerning the well-being consequences, both trait FOMO and state FOMO were positively correlated with feeling sleep-deprived (r = 0.31 and r = 0.31, *p* < 0.001), having difficulty in fulfilling daily responsibilities (r = 0.32 and r = 0.27, *p* < 0.001), lack of concentration during the day (r = 0.30 and r = 0.30, *p* < 0.001), having difficulty to enjoy the moment (r = 0.40 and r = 0.35, *p* < 0.001) and finding relief in the idea of others’ having a hard time keeping up with the digital content (r = 0.39 and r = 0.30, *p* < 0.001). These strong correlations suggest that participants with high levels of dispositional and situational FOMO still had trouble in following diverse digital content at this late stage of the pandemic, and experienced negative affective and physical consequences (see Table 2).

- FOMO and tendency to seek and share information:

Finally, at this stage, participants’ trait FOMO did not come up to be correlated with their eagerness to read more news or share what they read with others, upon checking the news about the pandemic (r = 0.09 and r = 0.06, *p* > 0.05). Even curious individuals did not show this tendency (r = 0.09 and r = 0.08, *p* > 0.05). In accordance, trait FOMO was positively correlated with increased time spent on social media, but not with following the news (r = 0.16, *p* < 0.05 and r = 0.09, *p* > 0.05). These results show that in the later stages of the pandemic, high levels of dispositional FOMO still related positively to spending more time on social media, but not to eagerness to follow or share the news. This was in stark contrast to our findings in early stage of the pandemic, when trait FOMO was positively related to individual information-seeking and sharing tendency. This suggests that over time, even high FOMO prone and curious individuals were likely to be fed up with and wanted to refrain from acquiring information on the pandemic (see Table 2).

## 3. Discussion

The COVID-19 pandemic has led to an inevitable surge in the use of digital tools and social media. With two studies conducted at the beginning and towards the end of the pandemic, we tried to uncover the triggers and the accompanying well-being effects of university students’ FOMO experiences resulting from this digital overuse.

Our results reveal that during the pandemic, even when socially distancing at home, individuals continued to experience FOMO. During this time, there has been a major shift in the type and amount of digital information individuals consumed. Our findings show that FOMO has been commonly experienced due to the difficulty of catching up with real-time social media content, others’ posts and videos, newly released movies and series on video-streaming platforms such as Netflix, and virtual gatherings with family and friends. Especially individuals who are more prone to FOMO as a personality characteristic reported feeling it more intensely about digital content. We find that like a vicious cycle, higher involvement in virtual activities feeds into experiencing higher levels of FOMO, which then leads to increased engagement with social media. Unlike commonsense would suggest, this difficulty of catching up with abundant digital content was not related to individual extroversion or productivity orientation as personality characteristics. Importantly, those with high levels of trait FOMO reported experiencing FOMO strongly on these virtual activities both at the peak and the late stage of the pandemic. There was no lessening of virtual FOMO even after the vaccine was announced and restrictions were loosened, suggesting that consumers may have been adapting to the highly digitalized “new normal” life.

Importantly, the only difference between the two periods was the tendency to seek and share information about the pandemic. Individuals who are more prone to FOMO, compared to their less-prone counterparts, reported being more eager to follow pandemic-related news and share what they read with others at the beginning, but not at the late stage of the pandemic. Even highly curious individuals did not want to seek out pandemic updates at the late stage. This is predictably due to individual desensitization to the pandemic news over time. The fact that FOMO proneness was strongly correlated with increased social media engagement at both stages, but not with following news at the late stage, suggests that people might have needed to focus away from pandemic updates and preferred the more interactive and entertaining consumption on social networking sites. We explored individual perceived risks of an acquaintance catching coronavirus as an additional variable in Study 1. Individuals who reported having a difficult time following pandemic updates also perceived it more likely that someone they knew could catch coronavirus. While we did not measure this relationship in Study 2, this finding suggests that experiencing FOMO towards reading pandemic related news might be positively related to individual risk perceptions of the disease.

Our data suggest that the increased consumption of digital content might have both affective and physical effects. Exploration of the well-being effects of this new form of FOMO during the pandemic revealed severe negative consequences. Due to experiencing FOMO, people report staying up at night later than usual due to a desire to catch up on digital experiences, which results in sleep deprivation, difficulty in focusing and enjoying the moment, as well as lower motivation to fulfill daily responsibilities. Most alarmingly, individuals who experience higher levels of FOMO indicate feeling better upon thinking that others might also be having difficulty catching up with the abundant digital content. As individuals try to make sense of their experiences, they find relief in imagining that others might be facing similar stresses—hardly a formula for a happy society. Again, these relations were consistent across the early and late stages of the pandemic, implying that our digital habits and the accompanied effects may linger even when the pandemic eventually recedes.

## 4. Conclusions

### 4.1. Our Contributions

The outbreak of COVID-19 as a worldwide health crisis has changed lives and routines around the world. One year into the pandemic, we still need time for the worldwide distribution of COVID-19 vaccine. Even in the areas where vaccines are available, there are people who are reluctant or refuse to get vaccinated, which delays the potential recovery from the pandemic. World Health Organization addresses vaccine hesitancy as one of the major health threats to global health [30]. While it is unknown how quickly we will reach herd immunity, both practitioners and scholars have been working hard to uncover and find ways to cope with the adverse effects of this pandemic at the individual and societal levels.

Responding to urgent calls to explore the well-being effects of the pandemic across disciplines [31], we specifically investigated the consequences of FOMO on individual daily affective and physical experiences in light of increased exposure to digital content. Staying indoors to flatten the curve has resulted in “quarantine fatigue,” as public health experts call it. Specifically, many find the abundance of virtual activities hard to keep up with, resulting in a new form of FOMO that puts a considerable burden on people’s well-being and happiness. Common sense would expect a repression and even disappearance of FOMO during a period when people are constrained in their physical movements and interactions. In contrast, we find that FOMO has not disappeared, but been replaced by a reshaped form of virtual FOMO experienced about online activities, resulting in important health concerns.

Previous research on FOMO has explored it as a feeling that occurs due to learning about other individuals’ attractive experiences that take place in the physical world, through digital channels (e.g., seeing friends’ vacation photos or party videos on social media). In other words, previous studies explored FOMO as a social feeling, resulting from comparing one’s situation with those of others. To our knowledge, no prior research had explored whether mere knowledge about existing online experiences such as Netflix movies and Instagram concerts also trigger FOMO. Our work shows that FOMO can be experienced towards attractive virtual activities, even in the absence of knowledge about other individuals’ (e.g., friends) involvement in these experiences. The abundance of digital content and the related feeling of difficulty to catch up with it are adequate to induce FOMO and related negative effects.

Our examination comes at a crucial time when it is still unclear when and how exactly we will recover from the COVID-19 pandemic. FOMO might be especially influential during this time due to the absence of engagements that have been shown to support subjective well-being and happiness, such as building intimate relations and socializing [32], work [33] and school engagement [34], spending time in nature [35], and taking group walks and exercising [36]. The pandemic has not only suspended these daily activities, but has created the new form of distanced living that has been severely affecting mental health by creating boredom, loneliness, sadness, stress, confusion, and anger (see [37] for a review).

Our results suggest that the public might need to recognize digital FOMO as an important and salient affective experience and find ways to manage and alleviate its effects. Some of the remote work and communication styles, as well as daily routines and avoidance behaviors that people have adopted, may remain permanent. Hence, recovering from the pandemic will be more than overcoming the virus. It will require mental and affective recovery. Looking at the past pattern of global pandemics such as SARS and the Spanish flu [38], similar physical distancing practices may be necessary in potential future pandemics, which stresses the importance of the topic under study. With virtual FOMO likely to be a continuing reality of life, questions remain on how to avoid its negative consequences and stay healthy during the pandemic and in the post-pandemic era.

### 4.2. Future Directions for FOMO and Its Impact on Well-Being

Experts have already started sharing tips on digital detoxing during the pandemic for mental health, such as restricting the excessive use of smart devices, setting daily routines to disconnect from the internet, or using social media specifically to seek useful and positive content rather than checking it out of habit or boredom [39]. Our results further point out the need to reduce the increased engagement with digital tools by highlighting its negative well-being effects among the younger generations. Given the difficulty in restricting the duration of someone’s accustomed digital engagement, disseminating information about the harmful effects of excessive screen exposure and internet use is particularly important to educate young people. Parents can encourage their children to be physically more active, maintain more regular sleeping habits and motivate enjoyable family activities such as playing games. Both public and school authorities can provide individuals with information about available psychological support services on internet addiction disorders as well as on coping with mental health effects of the pandemic. Policymakers can pressure digital technology companies to provide tools that send notifications to let users know the time duration spent online (such as on social networking sites or on digital apps) to help assess their exposure to digital devices. Seeing activity reports and time duration spent online can help individuals auto-control and limit their digital engagement. While many of the pandemic-induced digital habits may become permanent, it is unclear how eager people will be to get back to their old routines concerning their digital lives. It would be fruitful to explore if FOMO towards digital content is still experienced in the post-pandemic era, or if it will be replaced by FOMO towards offline experiences due to a digital saturation, and a desire to retrieve the face-to-face interactions with other people.

The recommendation to decrease social media engagement might not be an easy one to follow because this is a time period when people are in dire need of social interactions, and are dependent on digital and social media to fulfill that need. This relationship creates vulnerability to FOMO. One question worth exploring is whether active engagement such as posting and sharing content on social media—rather than passive engagement such as scrolling through and observing others’ profiles—could reduce FOMO. It is possible that being a poster rather than a lurker may actually prompt feelings of adequacy in being in control of and able to keep up with existing digital content, and offer a feeling of relief from FOMO.

Another path to decrease the anxiety of FOMO might be shifting people’s focus from others to themselves. Meditation and mindfulness practices have long been shown to enhance subjective well-being by orienting individuals towards a present moment [40]. Creating mindful awareness of the current moment may keep individuals from paying attention to concurrent other experiences. Relatedly, JOMO (the joy of missing out)—enjoying one’s current moment regardless of thinking about what others are doing [41]—is a popular term that has not been explored by previous academic work. It would be fruitful to explore the situational factors and incentives that might motivate enjoyment of the present—offline or digital experiences—while avoiding social comparisons with others. A shift from FOMO to JOMO might be helpful as people transition to the next stage in the pandemic.

Extant research has shown that time-related stress has serious negative effects on subjective well-being [42]. While many people have been busy with digital experiences during the pandemic, others have been conscious about enjoying the indoors and the slower life of not rushing into new activities. It has been ironic that while the pace of life slowed down in the physical world, it increased substantially in the digital. Higher exposure to mounting digital content may lead to feeling that time is more limited and elevate feelings of missing out. Accordingly, future research can explore the interplay between perceived time sufficiency and experiencing FOMO during digitally busy times.

Moreover, it might be fruitful for future work to explore whether being grateful for the things that one has and making the most of one’s present day—known as Carpe Diem [43]—may help with alleviating FOMO. During the pandemic, one perspective to adopt has been to perceive the quarantine days as a period for personal growth and productivity, and a reminder to be thankful for the simple moments and people one has around. Reminding the self and being appreciative of what one has is likely to prevent experiencing FOMO about unpursued experiences. It would be interesting to explore possible personality traits that might distinguish isolated living experiences, and individual adaptive responses during physically and psychologically restricting times.

Finally, as the vaccine is becoming available for some but not for everyone (February 2021), it would be interesting to examine whether people experience FOMO towards getting the vaccine and its potential psychological consequences. Stigma and discrimination are serious barriers to interact with people suspected of being infected in contagious diseases [44]. Given that it may take up to a few years until COVID-19 vaccine is fully distributed, a potentially interesting avenue for future research is to explore if an individual’s fear of missing out on vaccination may induce fear of being stigmatized and discriminated in society.

## Figures and Tables

**Table 1:** Descriptive statistics and relationships between FOMO and digital content. (Study 1 & Study 2).

Variables	Study	M	SD	1.	2.	3.	4.	5.	6.	7.
1. Trait FOMO	S1	3.74	1.09	1						
	S2	3.57	1.06	1						
2. State FOMO	S1	2.53	1.89	0.37 ***	1					
	S2	3.04	1.89	0.36 ***	1					
3. Actual engagement in virtual activities	S1	5.28	2.99	0.18 *	0.41 ***	1				
	S2	5.17	3.03	0.13 *	0.38 ***	1				
4. Real-time events (concerts, interviews, sports events)	S1	2.48	1.57	0.30 ***	0.30 ***	0.22 **	1			
	S2	2.78	1.76	0.34 ***	0.37 ***	0.22 ***	1			
5. Movies and series	S1	3.34	1.92	0.27 ***	0.20 **	0.11	0.43 ***	1		
	S2	3.67	1.97	0.33 ***	0.33 ***	0.14 *	0.48 ***	1		
6. Virtual gatherings with family and friends	S1	3.43	1.87	0.51 ***	0.26 **	0.21 **	0.41 ***	0.31 ***	1	
	S2	3.60	1.89	0.39 ***	0.42 ***	0.33 ***	0.45 ***	0.51 ***	1	
7. Others’ social media posts	S1	2.52	1.56	0.40 ***	0.35 ***	0.19 *	0.43 ***	0.31 ***	0.40 ***	1
	S2	2.72	1.72	0.43 ***	0.40 ***	0.17 *	0.60 ***	0.53 ***	0.50 ***	1
8. News on pandemic	S1	3.19	1.82	0.28 ***	0.19 *	0.18 *	0.27 ***	0.31 ***	0.35 ***	0.36 ***
	S2	3.18	1.85	0.13	0.29 ***	0.17 *	0.40 ***	0.40 ***	0.34 ***	0.42 ***

Study 1, *N* = 178; Study 2, *N* = 215; *** *p* ≤ 0.001, ** *p* ≤ 0.01, * *p* ≤ 0.05.

**Table 2:** Descriptive statistics and relationships between FOMO, well-being, and tendency to seek and share information. (Study 1 & Study 2).

Variables	Study	M	SD	1.	2.	3.	4.	5.	6.	7.	8.	9.	10.	11.
1. Trait FOMO	S1	3.74	1.09	1										
	S2	3.57	1.06	1										
2. State FOMO	S1	2.53	1.89	0.37 ***	1									
	S2	3.04	1.89	0.36 ***	1									
3. Sleep-deprivation	S1	3.29	2.22	0.29 ***	0.21 **	1								
	S2	3.03	2.11	0.31 ***	0.31 ***	1								
4. Difficulty in fulfilling daily responsibilities	S1	3.52	2.03	0.32 ***	0.23 **	0.62 ***	1							
	S2	3.12	1.94	0.32 ***	0.27 ***	0.71 ***	1							
5. Lack of concentration	S1	3.48	1.99	0.32 ***	0.18 *	0.65 ***	0.76 ***	1						
	S2	2.92	1.99	0.30 ***	0.30 ***	0.64 ***	0.80 ***	1						
6. Difficulty enjoying the moment	S1	2.93	2.00	0.44 ***	0.37 ***	0.50 ***	0.51 ***	0.56 ***	1					
	S2	2.60	1.81	0.40 ***	0.36 ***	0.58 ***	0.59 ***	0.61 ***	1					
7. Finding relief in others’ having a hard time	S1	2.74	1.94	0.47 ***	0.26 ***	0.35 ***	0.27 ***	0.34 ***	0.44 ***	1				
	S2	2.46	1.77	0.39 ***	0.30 ***	0.50 ***	0.51 ***	0.46 ***	0.53 ***	1				
8. Information seeking tendency	S1	4.28	1.96	0.34 ***	0.13	0.14	0.04	0.05	0.11	0.13	1			
	S2	4.20	1.82	0.09	0.23 *	0.13	0.07	0.13 *	0.07	0.11	1			
9. Information sharing tendency	S1	4.36	1.84	0.32 ***	0.20 *	0.13	-0.02	0.09	0.10	0.20 *	0.63 ***	1		
	S2	4.67	1.74	0.06	0.10	0.10	0.09	0.14 *	-0.01	0.07	0.65 ***	1		
10. Increased time spent on social media	S1	5.13	1.84	0.36 ***	0.16 *	0.16 *	0.22 *	0.23 *	0.24 *	0.17 *	0.47 ***	0.38 ***	1	
	S2	5.65	1.66	0.16 *	0.14 *	0.17 *	0.19 *	0.13	0.13	0.07	0.24 ***	0.17 *	1	
11. Increased time spent on reading news	S1	5.07	1.79	0.20 **	0.15 *	0.11	0.16 *	0.19 *	0.08	0.08	0.43 ***	0.50 ***	0.54 ***	1
	S2	4.90	1.95	0.09	0.16 *	0.15 *	0.07	0.06	0.06	0.13	0.29 ***	0.33 ***	0.39 ***	1

Study 1, *N* = 178; Study 2, *N* = 215; *** *p* ≤ 0.001, ** *p* ≤ 0.01, * *p* ≤ 0.05.

## Data Availability

All SPSS data files for the reported surveys will be made available at Dryad database (www.datadryad.org), and will also be shared with the reviewers during the review process upon request.
